# Exclusive breastfeeding: aligning the indicator with the goal

**DOI:** 10.9745/GHSP-D-14-00061

**Published:** 2014-08-05

**Authors:** Thomas W Pullum

**Affiliations:** a ICF International, The Demographic and Health Surveys Program, Rockville, MD, USA

## Abstract

While the global objective is exclusive breastfeeding (EBF) for a full 6 months duration, the standard indicator is a “prevalence” indicator, that is, the percentage of all children under age 6 months who are exclusively breastfed at a point in time. That yields a higher percentage than a more direct indicator of duration and can be easily misunderstood, exaggerating the amount of EBF. A measurement of actual percentage of children exclusively breastfeeding for a full 6 months can be easily calculated from standard DHS and MICS data.

Recognizing that exclusive breastfeeding (EBF) is a key to child survival, the World Health Organization (WHO) recommends that “infants should be exclusively breastfed for the first 6 months of life to achieve optimal growth, development and health.”^1^ To assess EBF, WHO, the United Nations Children's Fund (UNICEF),^2^^,^^3^ and the United States Agency for International Development (USAID)^4^ use an indicator defined as the percentage of children under 6 months of age who are being exclusively breastfed at a point in time. A recent and valuable report from WHO, *World Health Statistics 2013*, includes that indicator for most countries of the world under the label “exclusively breastfed **for** the first 6 months of life.”^5^

Thus, a discrepancy exists between the recommendation and the indicator. The programmatic recommendation is stated as a duration of EBF, but the indicator is stated as the prevalence of EBF in an age group at a point in time. To be more specific, the recommendation is that every child should be exclusively breastfed until reaching the 6-month anniversary of its birth, that is, for a duration of 6 months. The indicator, however, describes whether children under 6 months of age are currently being exclusively breastfed at the time that the survey is taken; in other words, it describes the prevalence of EBF. The recommendation and the indicator are misaligned. Moreover, the labeling of the indicator in the WHO report is ambiguous, depending crucially on the preposition “for,” and thus is easily misinterpreted.

The prevalence indicator yields much higher levels of EBF than a more direct indicator of duration would imply. For example, the WHO report gives an estimate of 52% for Ethiopia in the time interval 2005–2012. As explained below, Demographic and Health Survey (DHS) data can produce an indicator that measures actual duration. When applied to the Ethiopia data, this measure implies that the percentage of children who were being exclusively breastfed 6 months after their birth was 23%, less than half of the prevalence value of 52%.

It is useful to review the basic DHS data on breastfeeding. DHS surveys do not include a question such as “how long did you breastfeed [name]?” Whenever such a question has been asked, the responses are heavily heaped on multiples of 3 months, and especially multiples of 6 months, and thus are nearly useless for analysis. Instead, all DHS surveys use a current status question about the most recent child born in the past 36 months: “Are you currently breastfeeding [name]?” A “yes” response is followed by other questions on additional liquids or solid foods in the past 24 hours, making it possible to determine whether the child is being breastfed exclusively or with supplementary liquids or solids. The reference to the past 24 hours adds specificity, but it is of course possible that the child was given supplements at some earlier time and has moved in and out of the criteria for exclusive breastfeeding. The question is restricted to children who are living with the mother (the respondent) at the time of the survey, under the assumption that children not living with their mother are not being breastfed.

DHS reports the percentage of children who are being exclusively breastfed at the time of the survey for various age ranges. The Table provides some numbers that appear in the report on the Ethiopia 2011 DHS survey.^6^ For the age range 0–5 months (that is, less than 6 months) the percentage of children currently being exclusively breastfed is 52.0%. It is clear that this is the source of the figure of 52% for Ethiopia that is given in the WHO report.

**Table:** TABLE. Percentage of Children Currently Being Exclusively Breastfed (% EBF), by Elapsed Months (*a*) Between the Child's Month of Birth and the Month of Interview

***a***	**% EBF**	**n**
0–1	70.3	363
2–3	55.3	479
4–5	31.8	406
6–8	16.9	608
0–5	52.0	1,248

Numbers of children (n) are weighted. Limited to children who are living with the mother at the time of interview. Source: Ethiopia 2011 Demographic and Health Survey.

In another table in the same report, the median duration of exclusive breastfeeding is given as 2.3 months. The calculation of the median requires smoothing and interpolation, but the methods are not sophisticated.^7^ The same logic that underlies the calculation of the median duration of breastfeeding, and the same smoothing and interpolation techniques, could easily produce an indicator that would correspond to the WHO recommendation for 6 months of exclusive breastfeeding. Applying this approach to the Ethiopia 2011 DHS survey (and setting aside the matter of EBF interruptions noted above), it can be estimated that 23% of children had been exclusively breastfed for 6 months at the time of that survey.

Analysis of data from Ethiopia indicates that about 23% of children had been exclusively breastfed for 6 months at the time of the survey vs. 52% of children who were currently being exclusively breastfed.

Besides the DHS, the other major program of population-based surveys is UNICEF's Multiple Indicator Cluster Surveys (MICS). These surveys gather data on EBF in a very similar fashion to that of the DHS and could also generate the same indicator of the duration of EBF.

The extent to which EBF is reaching 6 months should be the indicator on center stage instead of the currently used prevalence indicator.

The currently used prevalence indicator has some advantages and has a legitimate role. Because it includes the entire sample of infants less than 6 months old, it has more statistical stability. Moreover, the health benefits of EBF are greater for the earlier months of life, and the prevalence indicator tends to give more weight to early EBF than the duration-to-6 months indicator. Still, if we want to promote 6 months of EBF, the extent to which EBF is reaching 6 months should be the indicator on center stage.

## Notes

**Peer Reviewed**

**First published online:** 2014 Aug 5
